# Alterations of the skin microbiome in HIV infection with pruritus

**DOI:** 10.3389/fcimb.2026.1749838

**Published:** 2026-04-22

**Authors:** Xue Ding, Leilei Fan, Xiuxia Ma, Jiahe Li, Pengyu Qian, Nao Qiu, Liran Xu, Jingyu Yue

**Affiliations:** 1 The First Affiliated Hospital of Henan University of Chinese Medicine, Zhengzhou, China; 2 The First People’s Hospital of Zhengzhou, Zhengzhou, China; 3 Henan University of Chinese Medicine, Zhengzhou, China

**Keywords:** 16S rRNA sequencing, HIV, HIV-associated pruritus, microbial biomarkers, skin microbiome

## Abstract

**Introduction:**

Pruritus is one of the main common symptoms of human immunodeficiency virus (HIV) infection. Skin changes caused by scratching, or the absence of skin findings despite ongoing pruritus, impact patients’ quality of life. With cutaneous HIV infection, pruritus is continuous, though it is unknown whether HIV infection affects the skin microbiota to cause pruritus.

**Methods:**

The skin microbiomes and plasma of HIV infection with pruritus, HIV and healthy were investigated in this study. Swabs were taken from four body sites and the composition of the microbiome at those sites was assessed using 16S rRNA amplification. Cytokines(interleukins 10 and 6) in plasma were detected by enzyme-linked immunosorbent assay.

**Results:**

The skin microbiome in the pruritus group was characterized by a significant depletion of protective commensals, specifically *Cutibacterium* and the *Burkholderia-Caballeronia-Paraburkholderia*. Conversely, opportunistic microbiome, including *Prevotella* and *Leptotrichia*, were markedly enriched and identified as key microbial signatures by Random Forest analysis. Correlation analysis revealed that the loss of protective commensals was positively associated with anti-inflammatory IL-10 levels, while the expansion of opportunistic pathogens was linked to elevated pro-inflammatory IL-6, indicating a microbial-driven immune imbalance.

**Conclusions:**

The results reveal that skin microbiota collapse and the loss of inherent anti-inflammatory defenses are pivotal features of HIV infection with pruritus.

## Introduction

During human immunodeficiency virus (HIV) infection, the incidence of delayed immune-mediated adverse reactions (mainly cutaneous) is up to 100 times (Chimbetete et al., 2023). Pruritus is a common symptom of HIV infection with pruritus; refractory pruritus can lead to scratching, picking, insomnia, and severe psychological stress. Causes of pruritus include skin infections, infections, ART reactions, papular squamous lesions, photodermatitis, Sjogren’s syndrome, and occasionally lymphoproliferative diseases (Singh and Rudikoff, 2003). HIV-induced immunologic dysregulation appears to lead to idioathic pruritus, but the precise mechanism is not fully understood. The itching signals are mainly transmitted through the following pathways: the small and unmyelinated C nerve fibers in the skin specifically stimulate the sensation of itch, cytokines produced by immune cells enhance the itch sensation, and they directly act on the sensory nerve fibers, triggering or intensifying the itch sensation (Bobotsis et al., 2024). Microorganisms in many parts of the body play an important role in homeostasis. Evidence indicates the important role of the skin microbiome in maintaining healthy skin and regulating skin-related diseases. The abundance and composition of the skin microbiome change during HIV infection (Ogai et al., 2023). HIV infection leads to immune dysregulation and systemic inflammation (Heron and Elahi, 2017). The skin barrier dysfunction caused by long-term immune activation may result in changes in microbial colonization patterns. Previous studies have shown that the skin microbiota is associated with pruritus in inflammatory skin diseases such as atopic dermatitis and psoriasis (Khadka et al., 2021; Chen et al., 2024). However, the microbiome and its relationship with inflammatory cytokines during HIV infection with pruritus remain unclear.

In this study, we collected skin microbiome samples and serum from people living with HIV(PLWH) with and without pruritus, as well as healthy, to assess the presence of dysbiosis and inflammation caused by immunosuppression. We selected interleukin-6 (IL-6) and interleukin-10 (IL-10) to evaluate the critical balance between pro-inflammatory and anti-inflammatory immune responses.IL-6 is a well-established driver of systemic immune activation and plays a pivotal role in the pathogenesis of pruritus (Oetjen et al., 2017). IL-10 is a pivotal immunoregulatory cytokine essential for mitigating tissue damage and maintaining skin homeostasis (Saraiva et al., 2020). By targeting this specific cytokine axis, we aimed to determine how host immune dysfunction and skin dysbiosis may relate to pruritus. Our findings should provide new insights for further studies to decipher the relationship between the skin microbiota in HIV infection and the development of pruritus.

## Materials and methods

### Study design

This was a part of a cross-sectional study.The inclusion criterion were including: (1) participants who were diagnosed with HIV, (2) visual analogue scale (VAS) (≥ 4) or not, (3) participants with pruritus who had not received systemic treatment in the past month. Healthy controls (aged 18–60 years) with no history of skin disease or current medical treatment were enrolled. All participants provided written informed consent prior to inclusion. Exclusion criteria included participants with serious diseases of vital organs, such as the heart, lung, liver, and kidney, or who had participated in drug clinical trials. The ethics committee of the First Affiliated Hospital of the Henan University of Chinese Medicine reviewed and approved the study protocol before enrollment (Approval No. 2025HL-497). Patients provided written informed consent before participating in the study.

### Sample collection

**Figure 1:**
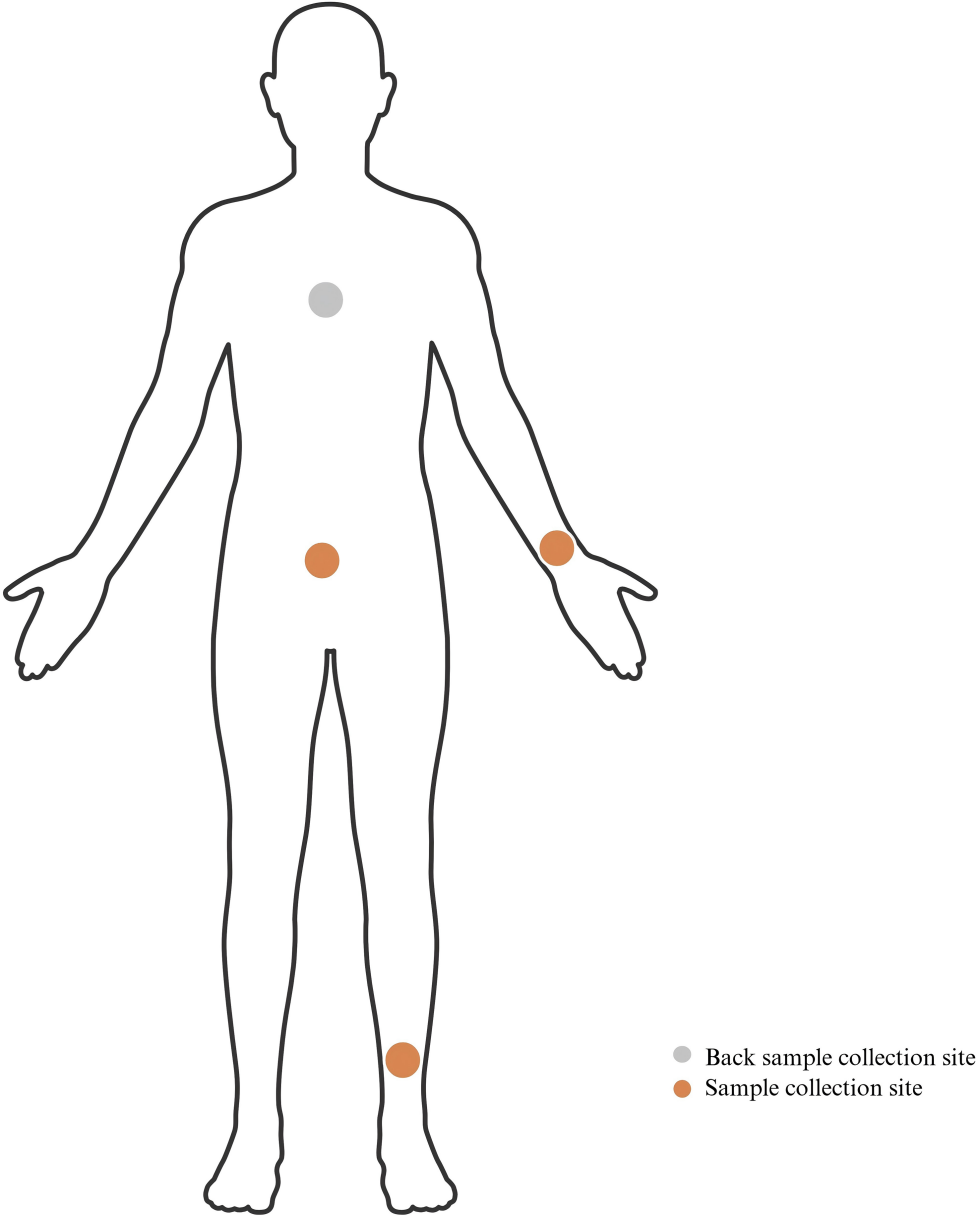
Sampled collection at four sites (righnterior forearm, lateral lower limbs, abdomen, back).

Superficial samples were taken from four sites to represent the range of commonly affected and unaffected sites: righnterior forearm, lateral lower limbs, abdomen, back (**Figure 1**). Skin swabs were collected as described previously (Ogai et al., 2022). Briefly, a swab was presoaked with normal aline, and designated positions of approximately 5 × 5 cm^2^ were swabbed six to eight times to collect microbiome DNA. Each swab head was cut and placed in a cryogenic vial containing cryopreservation medium before storage at −80 °C until DNA extraction. Plasma samples were collected in the morning with ethylenediaminetetraacetic acid(EDTA) anticoagulant tubes. Samples were then centrifuged at 1,000 xg for 15 min, and supernatants were aliquotted into cryovials for storage at −80 °C.

### Cytokine

Cytokine level was measured using enzyme-linked immunosorbent assay(ELISA) kit (Proteintech, IL-6 and IL-10 ELISA Kit, USA) according to the manufacturer’s instruction.

### DNA extraction

Total genomic DNA from skin microbiome samples was extracted using CTAB, following the manufacturer’s instructions, and stored at -20 °C before further analysis. The quantity and quality of extracted DNA were measured using a NanoDrop NC2000 spectrophotometer (Thermo Fisher Scientific, Waltham, MA, USA) and agarose gel electrophoresis, respectively.

### 16S rRNA gene amplicon sequencing

PCR amplification of the bacterial 16S rRNA gene V3–V4 region was performed using primers 338F (5’-ACTCCTACGGGAGGCAGCA-3’) and 806R (5’- GGACTACHVGGGTWTCTAAT-3’). PCR amplicons were purified with Vazyme VAHTSTM DNA Clean Beads (Vazyme, Nanjing, China) and quantified using a Quant-iT PicoGreen dsDNA Assay Kit (Invitrogen, Carlsbad, CA, USA). After individual quantification, amplicons were pooled in equal amounts, and paired-end 2250 bp sequencing was performed using the Illumina NovaSeq platform with a NovaSeq 6000 SP Reagent Kit at Shanghai Personal Biotechnology Co., Ltd (Shanghai, China).

### Sequencing analysis

Microbiome bioinformatics were performed with QIIME2 2024.5 (Bolyen et al., 2019) with slight modifications according to the official tutorials. Briefly, raw sequence data were demultiplexed using the demux plugin, followed by primer cutting with the cutadapt plugin (Martin, 2011). Sequences were then quality filtered, denoised, and merged, and chimeras were removed using the DADA2 plugin (Callahan et al., 2016).

### Bioinformatics and statistical analysis

Sequencing data analysis was performed using QIIME2 and R software (v4.3.3). Based on the amplicon sequence variant (ASV) table generated in QIIME2, taxonomic assignment was conducted. After taxonomic assignment, sequences identified as *Chloroplasts* or *Mitochondria* were excluded from further analysis to avoid the influence of environmental or host DNA contamination. Subsequently, alpha and beta diversity indices were calculated using the filtered ASV table. To assess the statistical significance of differences in microbial community structure (beta diversity) among the groups, PERMANOVA was utilized. Venn diagrams were generated to visualize the shared and unique ASVs across different samples and groups, regardless of their relative abundance (Zaura et al., 2009) Random Forest analysis (RFA) was conducted to identify key microbial features discriminating the study groups. Finally, the functional profiles of the microbial communities were predicted using PICRUSt2 (Phylogenetic Investigation of Communities by Reconstruction of Unobserved States), mapped against the MetaCyc and KEGG databases.

### Correlation analysis of different microbiota and cytokines

Spearman’s rank correlation analysis was performed to evaluate associations between the top 20 genera and cytokines (IL-6 and IL-10) in the pruritus group. Correlation coefficients and FDR-adjusted *P*-values (*q*-values) were calculated in R, and the results were visualized using the “pheatmap” package. Significance was determined based on a threshold of *q* < 0.05.

### Statistical analysis

Statistical analyses were performed using R version 4.3.3. The Kruskal-Wallis test was applied to compare alpha diversity indices and taxonomic relative abundances across the non-pruritus, pruritus, and healthy groups. Beta diversity was evaluated using Bray-Curtis distances. Discriminative microbial features were identified usin RFA. Spearman’s rank correlation coefficient was calculated to assess associations between specific microbial genera and host cytokines.

Statistical significance was defined as *P* < 0.05. The Benjamini-Hochberg False Discovery Rate (FDR) method was utilized to adjust for multiple testing (q < 0.05). Additionally, pronounced biological trends associated with nominal significance (P < 0.05) prior to adjustment were retained for exploratory discussion.

## Results

### Baseline characteristics

The skin microbiota was analyzed across three groups:PLWH without pruritus (Non-pruritus, n=26), PLWH with pruritus (Pruritus, n=24), and healthy controls (Healthy, n=20). Characteristics of the participants is described in **Table 1**.

**Table 1:** Characteristics of the participants.

Characteristics	Healthy group (n=20)	Non-pruritus (n=26)	Pruritus (n=24)
Male sex-no.(%)	12	10	11
Age, median (range) - yr	50.05(32-60)	54.07(45-65)	54.07(45-63)
BMI, mean ± SD	22.85 ± 3.409	23.45 ± 3.25	20.63 ± 2.39
ART regimens - no. (%)			
NNRTI-based		17(65.38)	16(66.67)
PI-based	–	8(30.77)	6(25.00)
Unknown	–	1(3.85)	2(8.33)
CD4 lymphocyte count, median (range) -cells/mm^3^	–	390.93 (174-919)	367.55 (177-840)
CD8 lymphocyte count, median (range) - cells/mm^3^	–	779(328-1507)	689(392-1254)

ART, antiretroviral therapy; NNRTI, non-nucleoside reverse transcriptase inhibitor; PI, protease inhibitor.

### Skin microbiota composition

**Figure 2:**
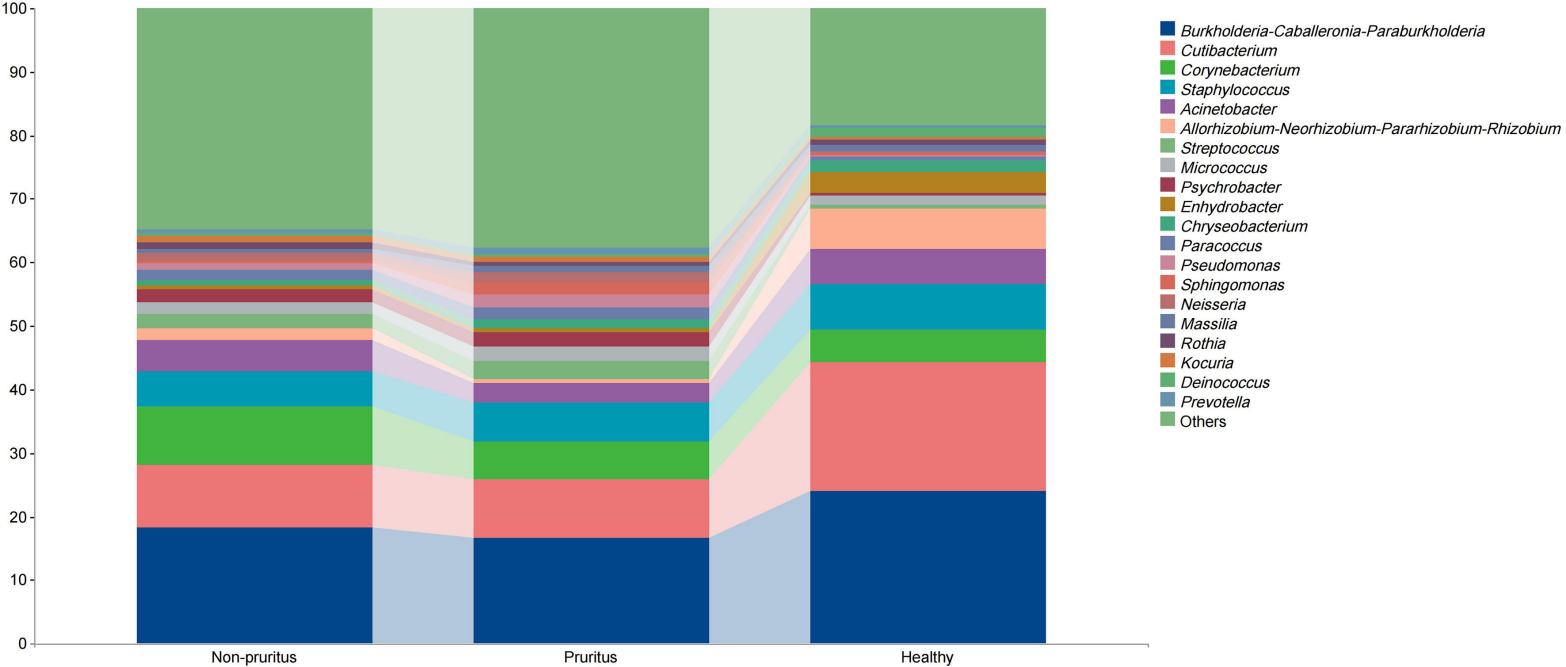
Relative abundance of the top 20 bacterial genera in the skin microbiota.

**Figure 3:**
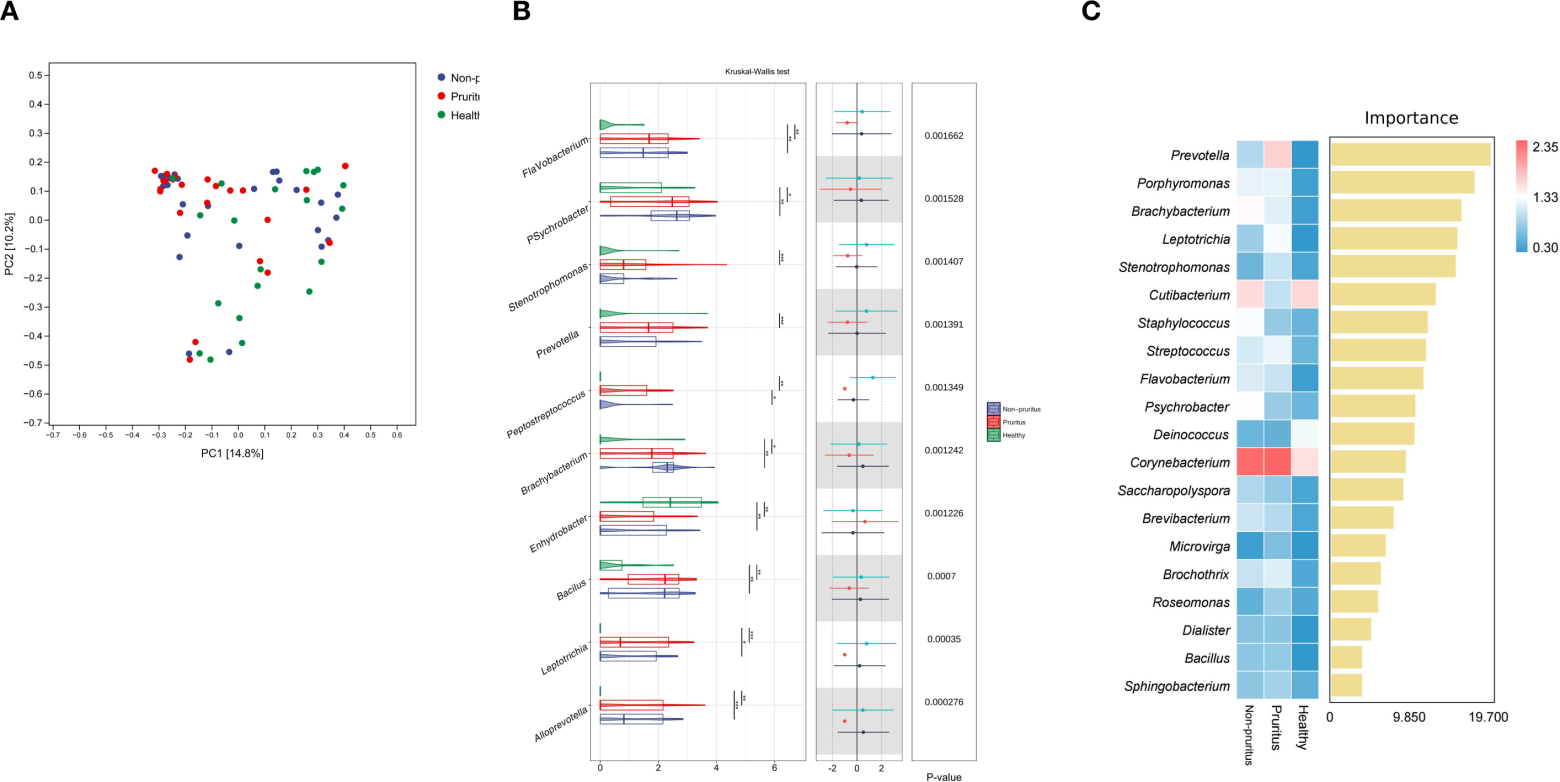
**(A)** PCoA of beta diversity based on Bray–Curtis distances. **(B)** Genus-level differences across the three groups evaluated by the Kruskal-Wallis test. The panel displays relative abundance box plots (left), effect size confidence intervals (middle), and P-values (right) **(C)** Random forest analysis reveals distinct microbial signatures among the three groups. Feature importance scores of the top 20 bacterial genera are shown on the right, with their corresponding relative abundance heatmap on the left.

The microbiome of the skin samples across the three groups contained > 20 genera primarily belonging to three phyla: *Proteobacteria, Actinobacteriota, and Firmicutes*. At the genus level, the most abundant sequences belonged to *Burkholderia-Caballeronia-Paraburkholderia*(BCP)*, Cutibacterium, Corynebacterium* (**Figure 2**). Notably, the relative abundances of BCP*, Cutibacterium*, and *Acinetobacter* in the pruritus group were noticeably decreased compared with those in the healthy group. The compositions of the skin microbiome in the three groups are shown in **Supplementary Table 1**. The alpha diversity indexes (total observed species, Chao1, Good’s coverage, Simpson’s index, Pielou’s e, Faith’s pd, Shannon, observed species, Allen’s H, and Rao’s quadratic entropy) in the pruritus group were not significantly different from the Non-pruritus and Healthy groups (**Supplementary Figure 2**). Beta diversity analysis using the Bray–Curtis distance revealed significant differences in the composition of the bacterial community between the three groups (**Figure 3A**).

To identify the significant differences in microbial composition among the three groups, a Kruskal-Wallis test was conducted at the genus level. The results revealed that the skin microbiota of the pruritus group was characterized by a significant enrichment of several specific genera. Notably, the relative abundances of *Alloprevotella, Leptotrichia, Stenotrophomonas, Peptostreptococcus*, and *Prevotella* were significantly increased in the pruritus group compared to the non-pruritus and healthy groups. (**Figure 3B**).

RFA was performed to identify the key microbial features discriminating the three study groups. The results demonstrated that *Prevotella, Porphyromonas, Brachybacterium, Leptotrichia*, and *Stenotrophomonas* exhibited the highest importance scores, indicating their roles as critical microbial signatures capable of distinguishing among the groups (**Figure 3C**). As shown, anaerobic bacteria such as *Prevotella* and *Porphyromonas*, as well as classic skin colonizing bacteria such as *Staphylococcus* and *Corynebacterium*, were less abundant in the healthy group. However, these microbial were significantly rich in the pruritus group. This imbalance of the microbiota may be caused by barrier damage and systemic immune activation, and is associated with HIV infection and pruritus symptoms.

### Functional prediction of the skin microbiome

To further determine the functional impact of skin microbiota alterations in HIV infection with pruritus, we predicted KEGG pathways using PICRUSt2. Functional prediction showed that metabolic pathways were the most abundant pathways predicted (**Supplementary Figure 2D**).

### Correlations between different intestinal microbiota and cytokine

**Figure 4:**
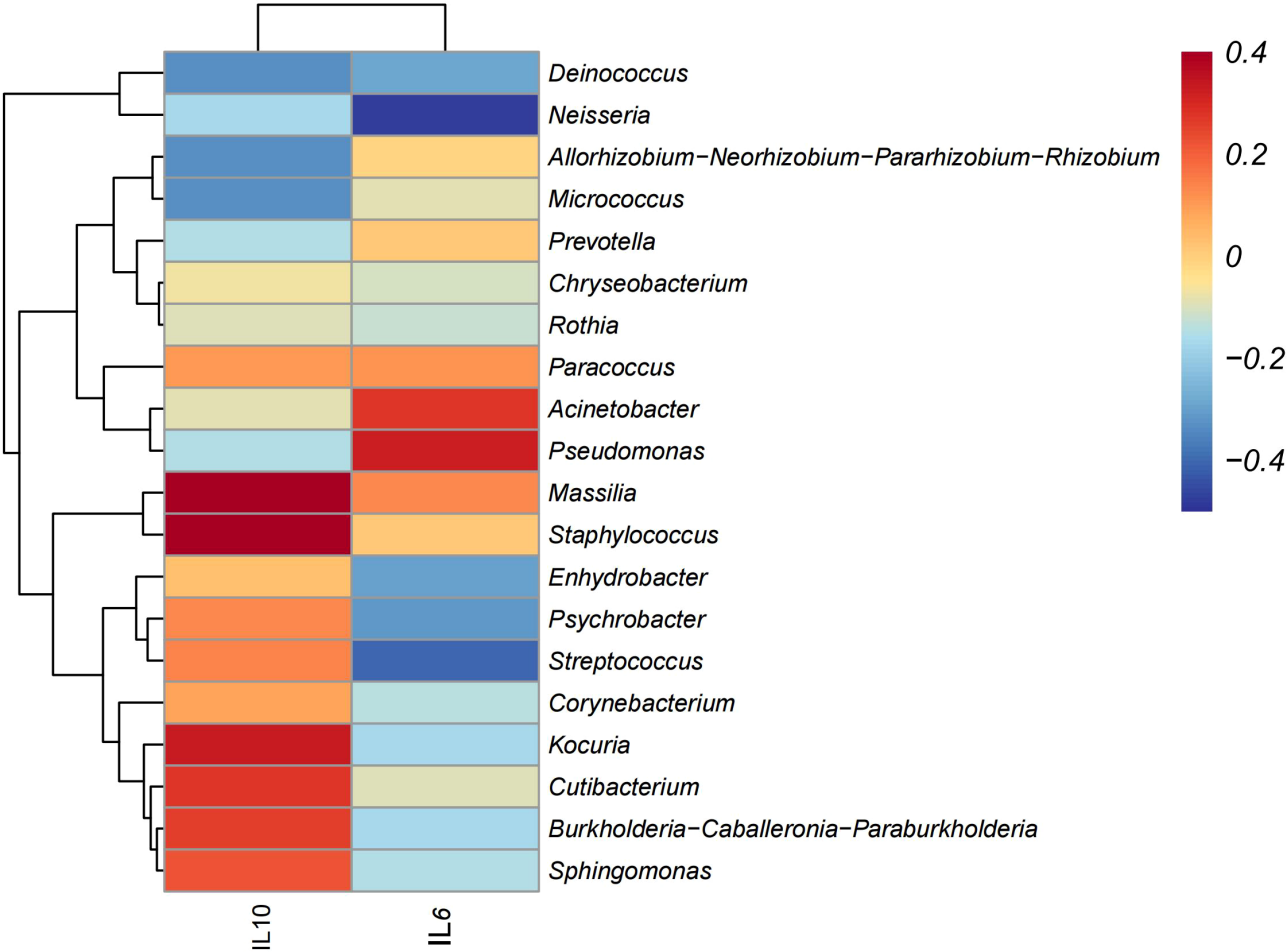
Spearman correlation heatmap between the relative abundance of the TOP 20 genera and cytokines (IL-10, IL-6). The color gradient denotes the Spearman correlation coefficient (R-value). No statistically significant correlations were observed after Benjamini-Hochberg (FDR) multiple testing correction (q > 0.05).

To investigate the potential interplay between the core microbiome and immune responses, a Spearman correlation analysis was performed between the top 20 dominant microbial genera and cytokines (IL-10 and IL-6) in the pruritus group. The correlation heatmap revealed distinct associative trends (**Figure 4**). Notably, genera such as Massilia, Staphylococcus, and Kocuria exhibited pronounced positive trends with IL-10. Similarly, Acinetobacter and Pseudomonas displayed strong positive associations with IL-6. Conversely, potential inverse relationships were also observed, with Neisseria and Streptococcus showing negative correlative trends with IL-6. Following Benjamini-Hochberg FDR correction to control for multiple testing, these associations remained no longer statistically significant(adjusted P > 0.05). This suggests that the local cytokine profiles may be modulated by broader, complex microbial community networks or synergistic metabolic interactions, rather than being solely driven by the abundance of a single genus.

## Discussion

Previous studies have investigated skin microbiome changes in people living with HIV. However, they have not been sufficient to fully elucidate the pathogenesis of pruritus. Our results reveal a progressive and stepwise alteration in the skin microbial landscape across the study groups, with the most severe microbiota collapse observed in the pruritus group. This trend indicates that while HIV-induced immune impairment creates microbial instability, the chronic mechanical trauma from repeated scratching plays a decisive role in driving the severe dysbiosis characteristic of itchy skin (Byrd et al., 2018).

In our study, a significant depletion of protective commensals was a defining feature of the pruritus group, specifically *Cutibacterium* and the BCP complex (*Burkholderia-Caballeronia-Paraburkholderia*). *Cutibacterium* is well-known for maintaining the skin barrier by converting host glycerol into short-chain fatty acids, thereby inhibiting pathogen colonization (Shu et al., 2013; Wollenberg et al., 2014). Our analysis showed these genera were most abundant in healthy groups but markedly reduced in Pruritus. Furthermore, our correlation heatmap revealed that both *Cutibacterium* and the BCP complex exhibited a positive biological trend with the anti-inflammatory cytokine IL-10, suggesting that their depletion compromises the skin’s inherent anti-inflammatory defenses and increases susceptibility to inflammatory triggers (Lee et al., 2025). The significant reduction in the abundance of these functional bacteria in the pruritus group resulting in the impairment of cutaneous antimicrobial defense systems. The interplay between skin microbial dysbiosis and host immune responses is a pivotal driver of HIV-associated pruritus, yet this area remains under-investigated (Dagnelie et al., 2022).

Complementing this loss of commensals, our Random Forest and Kruskal-Wallis analyses identified a pronounced expansion of opportunistic and atypical bacteria, including *Prevotella, Leptotrichia, Bacillus*, and *Peptostreptococcus*—within the Pruritus cohort. Notably, *Prevotella* and *Leptotrichia* emerged as the most discriminative features for pruritus. These bacteria are typically associated with mucosal or anaerobic environments and are non-resident on healthy skin, their proliferation underscores a profoundly breached skin barrier (Deng et al., 2023). Such a shift highlights a profound reorganization of the microbial community structure in patients with pruritus, as opposed to the simple overgrowth of an individual isolate.

Although individual microbe-cytokine associations did not survive rigorous FDR correction, pronounced biological trends remained evident. Specifically, the positive correlations between opportunistic pathogens (e.g., *Acinetobacter* and *Pseudomonas)* and the pro-inflammatory cytokine IL-6 suggest a functional link between dysbiosis and systemic inflammation. Chronic scratching mechanically disrupts the stratum corneum, allowing this dysbiotic community to penetrate deeper epidermal layers (Byrd et al., 2018). This microbial translocation, coupled with the loss of protective commensals such as *Cutibacterium*, triggers localized immune activation that likely contributes to the broader systemic elevation of IL-6 (Shu et al., 2013). Ultimately, the observed dysbiosis functions as a key bridge linking skin barrier failure to inflammatory pruritus. Considering the impact of lifestyle, environmental and other factors on the skin microbiome,further researches are needed to explore the relationship between the microbial changes and the pathogenesis of pruritus.

In conclusion, our study reveals changes in the skin microbiome in patients infected with HIV with pruritus. The results provide fundamental information for understanding the mechanism behind pruritus development in HIV infection.

## Data Availability

The datasets presented in this study can be found in online repositories. The names of the repository/repositories and accession number(s) can be found below: https://www.ncbi.nlm.nih.gov/genbank/, PRJNA977211.
