# Supplementary figures and images for: Alterations of the skin microbiome in HIV infection with pruritus
Source: Front Cell Infect Microbiol. 2026 Apr 22;16:1749838. doi: 10.3389/fcimb.2026.1749838 (PMC13144027; doi:10.3389/fcimb.2026.1749838)

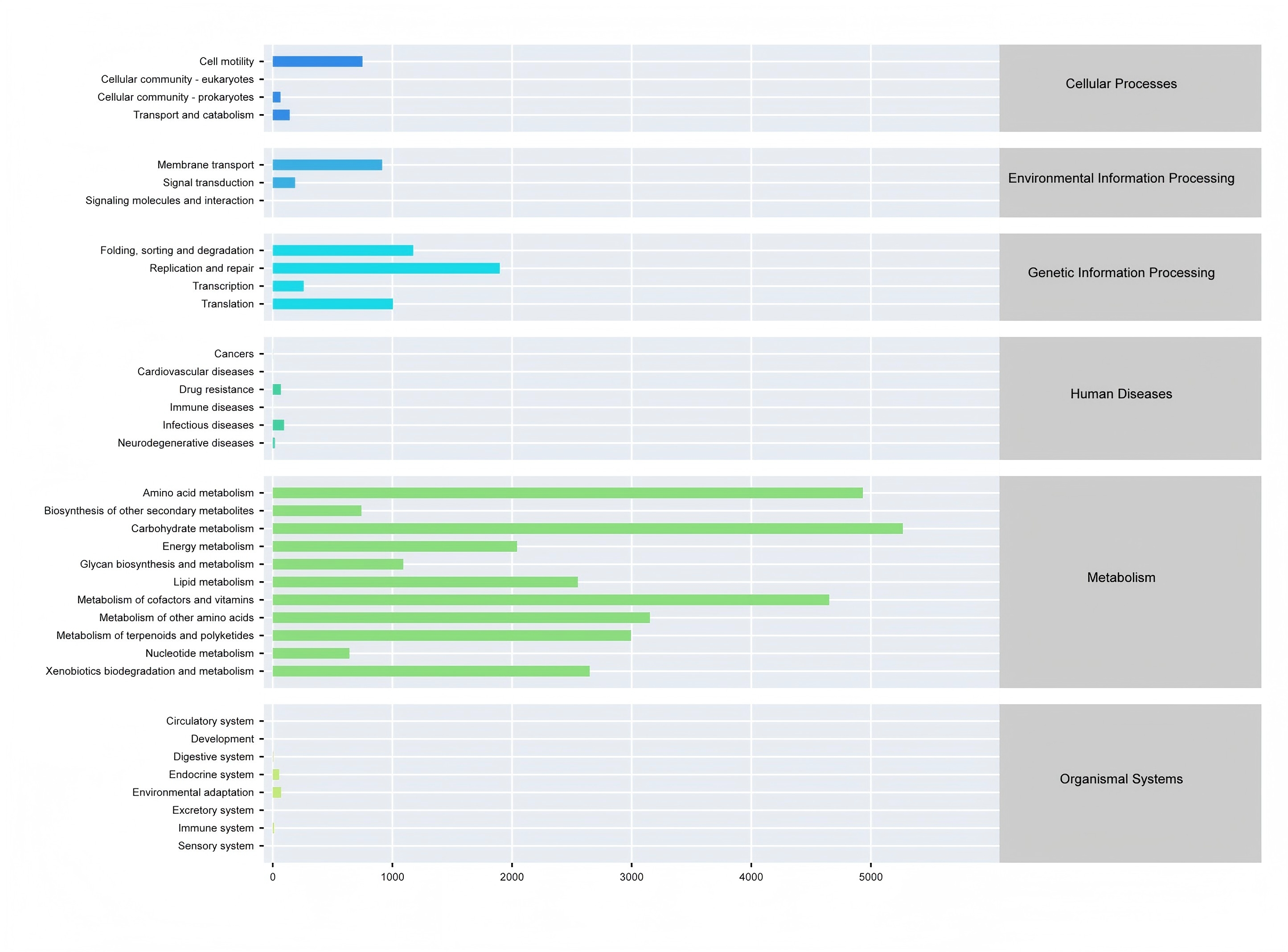

Supplement: Supplementary file 1 [file Image1.jpeg]

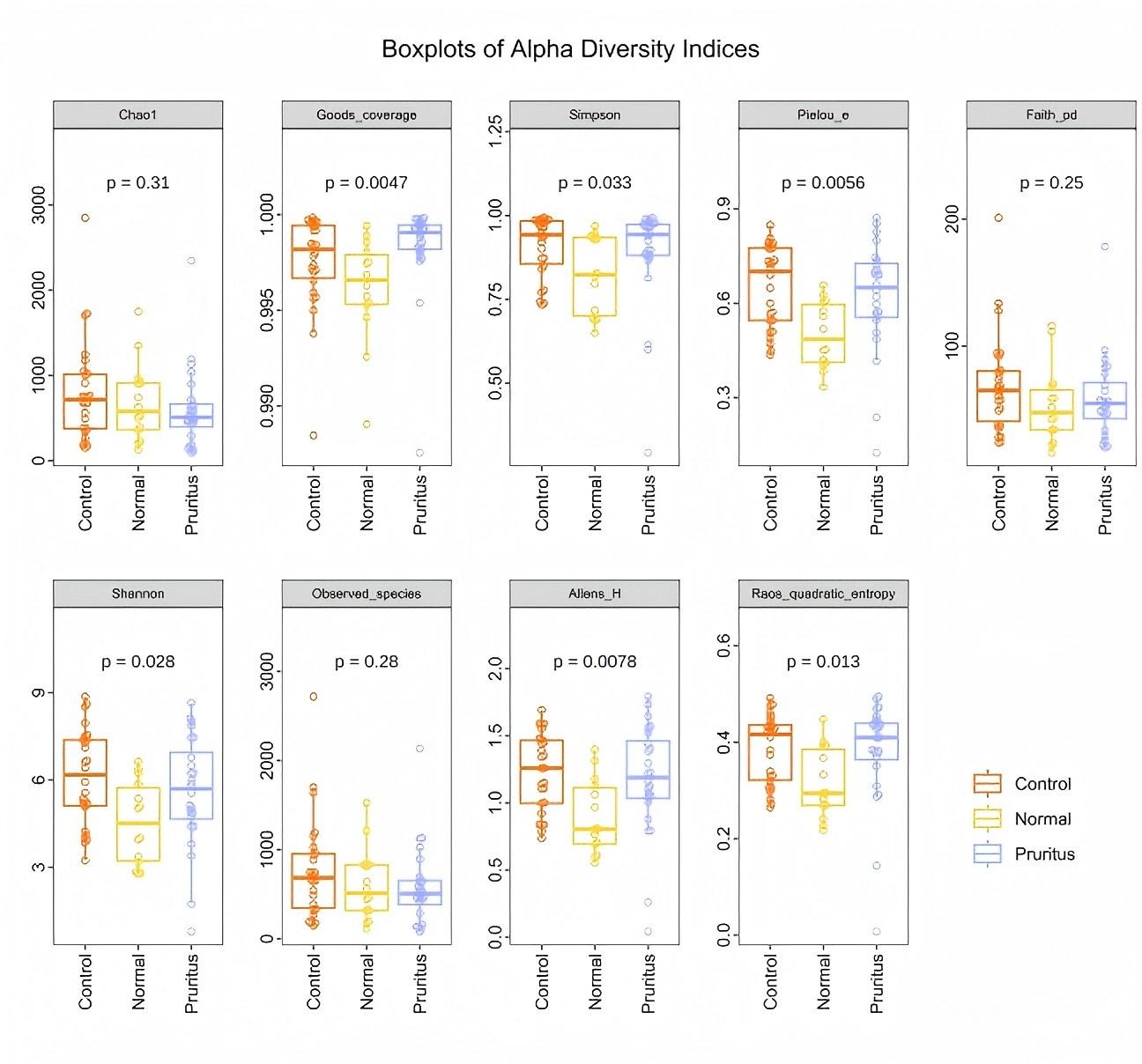

Supplement: Supplementary file 2 [file Image2.jpeg]
